# Thermal Properties of Surface-Modified and Cross-Linked Boron Nitride/Polyethylene Glycol Composite as Phase Change Material

**DOI:** 10.3390/polym13030456

**Published:** 2021-01-31

**Authors:** Jaehyun Wie, Jooheon Kim

**Affiliations:** 1 School of Chemical Engineering & Materials Science, Chung-Ang University, Seoul 156-756, Korea; grwie23@naver.com; 2 Department of Intelligent Energy and Industry, Chung-Ang University, Seoul 156-756, Korea

**Keywords:** PCM, boron nitride, thermal conductivity, polymer composite, in-situ polymerization

## Abstract

A thermally conductive phase change material (PCM) was fabricated using polyethylene glycol (PEG) and boron nitride (BN). However, the interfacial adhesion between the BN and the PEG was poor, hindering efficient heat conduction. Grafting polyvinyl alcohol (PVA) onto the surface of BN and cross-linking due to hydrogen bonding between the hydroxyl groups in PVA and oxygen atoms in PEG improved the wettability of fillers. By employing this strategy, we achieved a thermal conductivity value of 0.89 W/mK, a 286% improvement compared to the thermal conductivity of the pristine PEG (0.23 W/mK). Although the latent heat of composites decreased due to the mobility of the polymer chain, the value was still reasonable for PCM applications.

## 1. Introduction

Phase change materials (PCMs) are substances that preserve and release thermal energy during the phase change process within a narrow temperature range. PCMs have been used for thermal energy storage and management. PCMs have high-energy density and nearly isothermal behavior [1]. High-energy density is a desirable property for energy storage systems. Thus, PCMs have been used in various energy storage applications such as solar panels, waste heat restoration, and other heat energy storage systems [2].

Among the many different PCMs, polyethylene glycol (PEG) is a widely used polymer PCM due to its moderate phase change temperature, nontoxicity, and high latent heat [3]. However, PEG has a low thermal conductivity and weight leakage during phase transition (0.2~0.3 W/mK) [4,5]. This low thermal conductivity is disadvantageous for energy storage applications. To overcome this problem, many different studies have been conducted [6,7,8]. Various thermal conductive fillers have been applied to improve the thermal conductivity, including carbon-based [9,10,11], ceramic [12,13], and metallic fillers [14,15]. Specifically, boron nitride (BN) is a universal ceramic filler for thermally conductive composites [16,17,18] because BN is highly thermally conductive and electrically insulated, which are desirable properties for thermal interface materials [19,20].

However, BN is a nonpolar substance, resulting in poor interfacial adhesion with PEG, which can cause crack formation and air voids in polymer composites. Phonon scattering can be caused and hindered by creating thermal boundary resistance. This negatively affects the conductive and mechanical properties of the polymer composites [21,22].

Polyvinyl alcohol (PVA) is a highly hydrophilic substance because it has many hydroxyl groups in its main chain. This polymer is produced through a polymerization and conversion process. Vinyl acetate is polymerized to make polyvinyl acetate (PVAc) and then converted into PVA [23].

In this study, BN, an excellent thermally conductive filler, was selected to increase the thermal conductivity of PEG with low thermal conductivity. However, the poor compatibility between the BN and the PEG results in inefficient heat conduction. In this work, we grafted PVA onto the surface of nonpolar BN to improve its compatibility with PEG. In addition, hexamethylenediamine, which was used as a cross-linker, was added to induce hydrogen bonding between PEG and PVA-grafted BN. In this paper, the composite through all these processes is defined as P-c-BN/PEG. After surface modification and cross-linking, the heat transfer efficiency was improved, enhancing the thermal conductivity. At the 30 wt.%, the thermal conductivity of P-c-BN/PEG was 0.89 W/mK, which was improved 286% higher compared to the neat PEG. The latent heat value, which is important for PCMs, decreased by 22% because the free volume of the polymer chain decreased due to improved interfacial adhesion and cross-linking. However, the value (137.5 J/g) was still acceptable for PCM applications.

## 2. Experimental

### 2.1. Materials

Boron nitride was obtained from LG Innotek, Seoul, South Korea. Polyethylene glycol 4000, ethanol (99.5%), sulfuric acid, H_2_SO_4_ (≥99%), potassium permanganate, and KMnO_4_ (≥99%, powder of crystals) were purchased from Daejung Chemicals [24]. Vinyltriethoxysilane (97%), vinyl acetate (≥99%), potassium persulfate, K_2_S_2_O_8_ (≥99.0%, powder of crystals), phosphorus pentoxide, P_2_O_5_ (99%, powder), acetic acid (≥99%), ammonium hydroxide, hydrogen peroxide, and H_2_O_2_ (30 wt.% in H_2_O) were purchased from Sigma Aldrich Korea.

### 2.2. Preparation of BN-OH

BN (10 g) was mixed with H_2_SO_4_ (40 mL), K_2_S_2_O_8_ (5 g), and P_2_O_5_ (5 g) at 80 °C for 4.5 h. The solution was cooled until the temperature reached 20 °C. Then, 1 L of distilled water was poured into the solution and left overnight. Then, the solution was washed with vacuum filtration to adjust to pH 7~8. The BN was obtained after drying in a convection oven at 50 °C overnight. As-prepared BN (30 g) was mixed with H_2_SO_4_ (400 mL) and KMnO_4_ (30 g) for additional oxidation. This solution was heated for 2 h at 35 °C. Then, 1 L of distilled water was added and the solution was mixed for 2 h. Finally, 20 mL of 30 wt.% H_2_O_2_ was poured into the solution. After this step, the solution became white in color. This solution was washed with 10 wt.% HCl and distilled water until the pH was about 7~8. BN-OH was obtained after drying in a convection oven overnight.

### 2.3. Preparation for Grafting Vinyltriethoxysilane on BN (VTES-BN)

BN was functionalized with vinyltriethoxysilane (VTES) via the hydrolyzation and condensation of VTES. Then, 10 g of BN-OH was dispersed in ethanol (350 mL) and distilled water (150 mL). Then, VTES (5 mL) was added to distilled water (20 mL) and the pH of the solution was adjusted to 4~5 using acetic acid at 50 °C for 30 min. Hydrolyzed VTES was added to the BN-OH solution and ammonium hydroxide was added to adjust the pH to 9~10 to condense the VTES and the hydroxyl group of BN-OH. After these steps, the solution was washed with ethanol three times and dried in a convection oven at 50 °C overnight.

### 2.4. PVA Grafting

PVAc was synthesized via the polymerization of vinyl acetate. Commercial soap (0.025 g), potassium persulfate initiator (0.25 g), vinyl acetate (5 mL), and VTES-BN (10 g) were mixed in distilled water (500 mL) at 80 °C for 1 h. To alcoholize PVAc, PVAc-BN (10 g) and 1 M NaOH (80 mL) were dispersed in methanol (400 mL).

### 2.5. Fabrication of PEG Composites

PVA-BN, hexamethylenediamine, and PEG were mixed in ethanol at 80 °C until the ethanol was evaporated. After evaporation, the mixture was poured into a Teflon mold and dried at room temperature overnight.

### 2.6. Characterization

Fourier-transform infrared (FTIR) spectra were collected on a Spectrum One spectrometer (PerkinElmer, Waltham, MA, USA). X-ray photoelectron spectroscopy (XPS) was conducted to analyze the PSZ and silane coating on the A-BN composite using an ESCA 2000 XPS (VG Microtech, London, UK). The water contact angle was directly measured using a contact angle goniometer (Rame-Hart, 100-00-(115/220)-S). The X-ray diffraction (XRD, Karlsruhe, Germany) patterns were collected with a new D8-Advance (Bruker-AXS) instrument at a scan rate of 1° s^−1^ with a 2θ range of 15°–70° and Cu Kα1 radiation (0.154056 nm). Composition of the samples was investigated by thermogravimetric analysis (TGA, TA Instruments, TGA-2050) at a heating rate of 10 °C min^−1^ in nitrogen flow. A Sigma field emission scanning electron microscope (FE-SEM, Carl Zeiss, Oberkochen, Germany) [25] was used to investigate cross sections of the composites and surface of the filler. The thermal diffusivities of disk-shaped samples were measured via laser flash analysis (LFA) at room temperature using a LFA 467 NanoFlash light flash apparatus (Netzsch, Selb, Germany). With the specific heat capacity (*C*_p_) at 20 °C, latent heat was measured via differential scanning calorimetry (DSC) using a DSC-7 differential calorimeter (PerkinElmer, USA). The bulk densities of the composites were measured using the water displacement method. The thermal conductivity of each sample was calculated by multiplying its density and *C*_p_ by its thermal diffusivity.

## 3. Results and Discussion

### 3.1. Grafted Fillers’ Analysis

#### 3.1.1. Water Contact Angle and FTIR

The water contact angle (WCA) was measured to investigate the hydrophilicity of the fillers. The filler was fabricated into a pellet using a hot press. Then, water droplets were dropped onto the surface of the filler pellet. The WCA of raw BN and PVA-BN was about 74° and 57.2°, respectively. Water permeated through the filler pellet easily after PVA grafting, which decreased the WCA. This decrease was due to the hydroxyl groups in the PVA.

**Figure 1:**
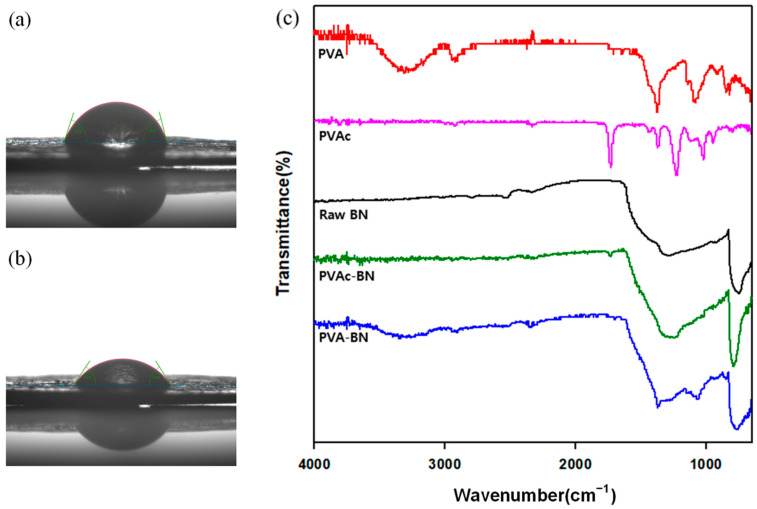
Surface analysis of raw BN and modified BNs. (**a**) Water contact angle of raw BN, (**b**) water contact angle of PVA-BN, (**c**) FTIR spectra of fillers.

FTIR curves of the samples are shown in Figure 1c. Raw BN had characteristic peaks representing the out-of-plane B-N-B bending vibration at 820 cm^−1^ and the in-plane B-N stretching vibration at 1360 cm^−1^. Peaks at 1728, 1380, and 1220 cm^−1^ were detected in PVAc-BN, and were also in the raw PVAc curve. Characteristic peaks of O–H, C–OH, C–H appeared in the PVA curve. These peaks were also present in the PVA-BN at 3400 (O–H), 2950, 1380 (C–H), 1090 cm^−1^ (C–OH).

#### 3.1.2. XPS

**Figure 2:**
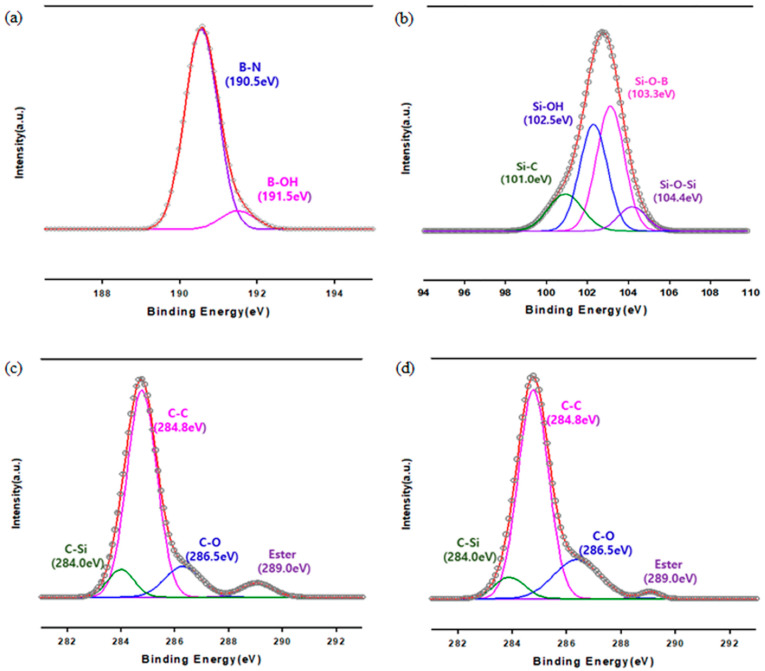
XPS spectra of fillers. (**a**) Hydroxylated BN (BN-OH), (**b**) VTES-BN, (**c**) PVAc-BN, (**d**) PVA-BN.

XPS results are shown in Figure 2. The B-OH peak detected at 191.5 eV formed from the oxidation process, which bridged the BN and VTES. In addition, four fitting peaks were observed in the Si 2p deconvolution of VTES-BN. Peaks at 101.0 eV, 102.5 eV, 103.3 eV, and 104.4 eV corresponded to Si–C, Si–OH, Si–O–B, and Si–O–Si, respectively [26]. The presence of Si-O-B confirmed bonding between the BN and VTES. The XPS results of PVAc-BN and PVA-BN are also depicted in Figure 2c,d, which were similar because PVA and PVAc have similar structures. Both spectra exhibited C–O (286.5 eV), C–Si (284.0 eV), and ester group (289.0 eV) peaks [27]. However, the intensity of the C–O and ester group peaks changed when PVA was converted into PVAc because PVA has many more C–O groups. This peak change suggests the successful conversion of PVAc into PVA.

#### 3.1.3. TGA and Morphology

TGA was used to investigate the surface-modified BN quantitatively. Raw BN was thermally stable such that it did not lose weight until the temperature reached 800 °C. However, the weight of VTES-BN started to decrease after 270 °C. The decomposition of VTES-BN caused the weight loss [28]. In addition, significant weight loss occurred in the case of the PVAc-BN and the PVA-BN.

**Figure 3:**
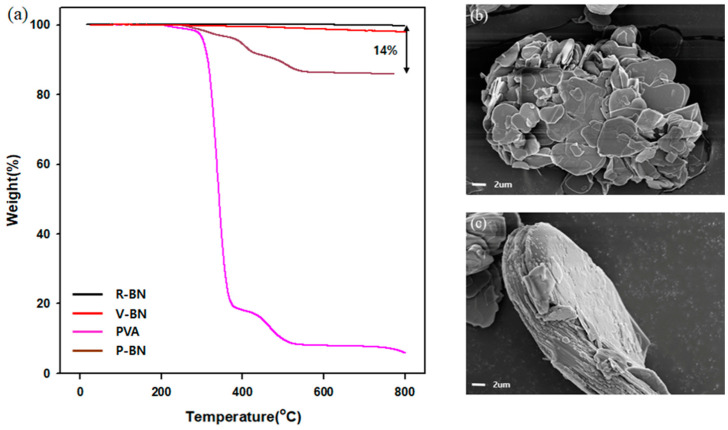
(**a**) TGA graph of fillers and FE-SEM image of fillers. (**b**) Raw BN, (**c**) PVA-BN.

The morphology of fillers was investigated through FE-SEM. Raw BN had a smooth surface, as shown in Figure 3b. However, the morphology of PVA-BN was different from the image of raw BN. Polymerized PVA covered the surface of BN in the case of PVA-BN.

#### 3.1.4. XRD

**Figure 4:**
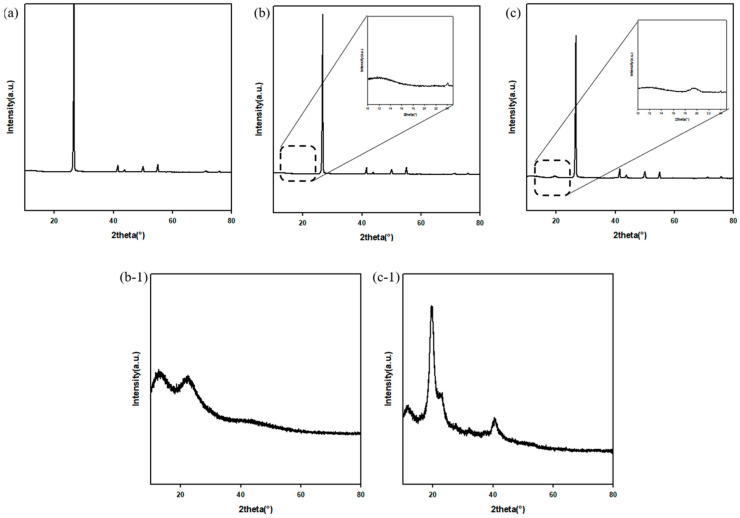
XRD graph of fillers. (**a**) Raw BN, (**b**) PVAc-BN, (**c**) PVA-BN, (**b-1**) PVAc, (**c-1**) PVA.

XRD was used to investigate possible changes in the crystal structures of BNs. As shown in Figure 4a, characteristic peaks such as (002), (100), (101), (102), (004), (110), and (112) were present. [29] PVAc-BN had no differences compared to h-BN because the raw PVAc only showed a weak peak around 2θ = 22.5° and this peak did not appear in the XRD graph of PVAc-BN. However, there was a different peak in the XRD graph of the PVA-BN. Raw PVA had a sharp peak around 2θ = 19.5°, which also appeared in the XRD graph of the PVA-BN. This was because the PVA was partially crystalline on formation [30].

### 3.2. Properties of the PEG Composites

#### 3.2.1. Morphology

**Figure 5:**
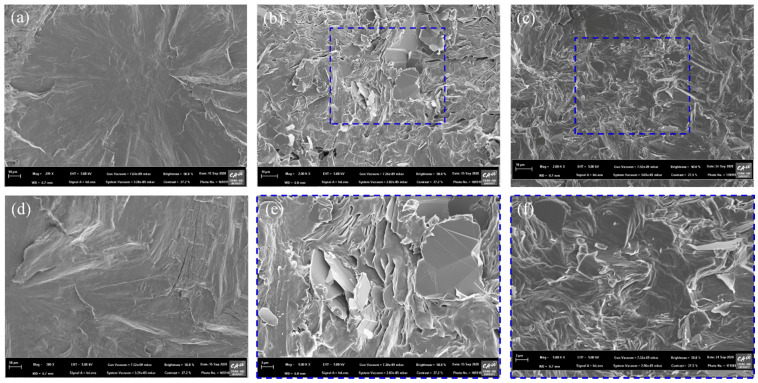
FE-SEM cross-section image. (**a**) PEG, (**b**) BN/PEG, (**c**) P-c-BN/PEG, (**d**) magnified image of PEG, (**e**) magnified image of BN/PEG, (**f**) magnified image of P-c-BN/PEG.

The morphologies of PEG composites were investigated by cross-sectional FE-SEM imaging. The neat PEG image exhibited a spotless and clean structure (Figure 5a). Figure 5b shows the image of the BN/PEG composite. The affinity between the BN and the PEG was investigated in these images. There were many voids and cracks because the raw BN had poor compatibility with the polar PEG. After PVA grafting and cross-linker addition onto the surface of the BN, the cross section of the PEG composite was dramatically changed. Hexamethylenediamine bridged the PEG and PVA grafted onto the BN via hydrogen bonding between the amine group and the hydroxyl group. This improved the interfacial adhesion between the filler and the matrix, thus reducing voids and cracks (Figure 5e).

#### 3.2.2. Thermal Properties

##### Thermal Stability

**Figure 6:**
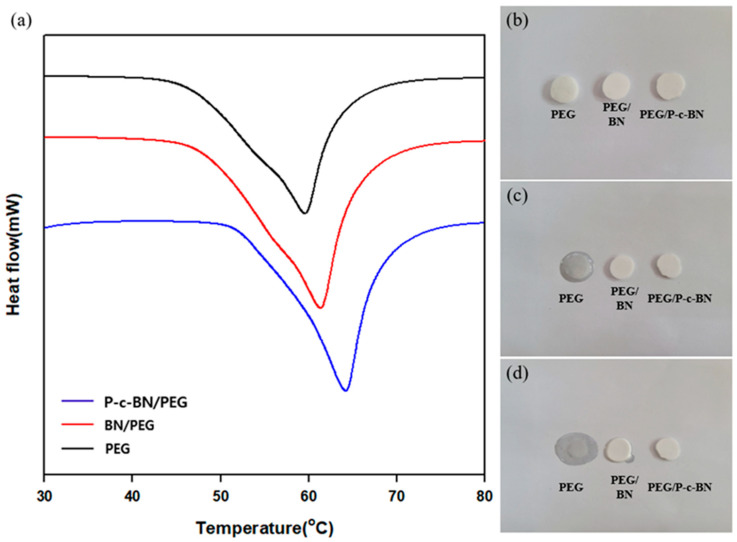
Thermal properties of composites. (**a**) DSC curve of composites. Composites’ heat on the hot plate for different times: (**b**) 0 s, (**c**) 120 s, (**d**) 240 s.

DSC was used to measure the thermal properties of the composites during heating. Endothermic peaks were observed around 60 °C due to melting (Figure 6a). The melting temperatures (T_m_) of the PEG composites were obtained from the endothermic curves measured by DSC. The T_m_ of the pristine PEG was around 60 °C. The T_m_ of the BN/PEG shifted approximately 2 °C higher. The incorporation of filler into the matrix increased the Tm of composites. The filler hindered the mobility of the polymer chains. In the case of P-c-BN/PEG, the hydrogen bonding between the hexamethylenediamine and the hydroxyl group of the PEG degraded the chain mobility of the polymer matrix. Thus, the polymer chain required extra heat for movement compared to samples without hydrogen bonding.

As mentioned in previous literature, the melting enthalpy(ΔH_m_) is important for PCMs such as PEG [31]. The ΔH_m_ of the pure PEG was 176.0 J/g and the ΔH_m_ value decreased to 152.6 J/g and 137.5 J/g for BN/PEG and P-c-BN/PEG, respectively. This decrease may have occurred due to steric effects, which changed the structure of the polymer chains. At temperatures above T_m_, the mobility and the free volume of the polymer matrix increased, which led to the decrease of ΔH_m_. This effect was related to the reduced T_m_ of the PEG/BN. Additionally, in the case of P-c-BN/PEG, the interactions between the filler and the matrix decreased the free volume of polymer chain. Significant interactions between the filler and the matrix did not occur due to the apolarity of raw BN. However, the increased polarity due to the PVA grafting onto the surface of BN generated hydrogen bonding, which decreased the free volume of the polymer chain. This can reduce ΔH_m_ according to Equation (1):

ΔH_m_ = β′ Δv (1)

where β′ is a constant and Δv is the variation in the free volume of the polymer [32].

Thermal stability is also important for PCMs. To investigate the thermal stability of our samples, samples were heated on a hot plate (Figure 6b). Conspicuous shape changes were not detected at temperatures below T_m_ (30 °C). However, raw PEG started melting at 80 °C and lost its shape. This phenomenon was more severe at low filler fractions. As the filler fraction increased, the steric effect hindered the mobility of polymer. However, in the case of P-c-BN/PEG, the composite maintained its shape well compared to other samples.

##### Thermal Conductivity

The thermal diffusivity of samples was measured using LFA. The thermal conductivity was obtained using the thermal diffusivity and Equation (1) [33].

*K* = *α ρ C*_p_, (2)

where *K* represents the thermal conductivity of the composite, *α* represents the thermal diffusivity of the composite, *ρ* is the density of the composite, and *C*_p_ is its specific heat capacity.

**Figure 7:**
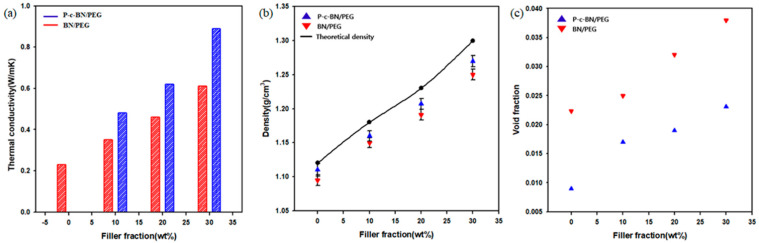
(**a**) Thermal conductivity of composites, (**b**) theoretically expected densities and experimental densities, (**c**) void fraction of fabricated composites.

Figure 7a shows the heat transferring performance of PEG composites. The thermal conductivity of the composites monotonically increased as the filler fraction increased. The thermal conductivity of PEG/BN composites at 10, 20, and 30 wt.% were 0.35, 0.46, and 0.61 W/mK, respectively. The thermal conductivity was improved 165% after addition of 30 wt.% of raw BN. The addition of the BN into the PEG built bridges between the polymer chains, and BN bridges improved the heat transfer in BN/PEG composites. This phenomenon occurs more frequently at high filler fractions. However, the compatibility between the BN and the PEG was poor. It can cause phonon scattering and decrease the heat transfer efficiency. This fact was confirmed by the FE-SEM image of the BN/PEG composite cross section, which showed the presence of cracks and air voids around the interface between the BN and the PEG matrix. Because the thermal conductivity of air is very low (thermal conductivity of air = 5 × 10^−5^) [34], voids and cracks can interrupt the heat flow path.

After PVA grafting and cross-linking, the voids and cracks were removed. The presence of polar PVA and cross-linking due to hydrogen bonding improved the affinity between the BN and the PEG. Thermal conductivity of the composites was 0.48, 0.62, and 0.89 W/mK at 10, 20, and 30 wt.% filler fraction, respectively. The thermal conductivity enhancement (TCE) from PVA grafting and cross-linking was 34, 31, and 52% for the 10, 20, and 30 wt.% filler fractions. At higher filler fractions, the amount of grafted PVA and the number of cross-linked particles was higher. Thus, the TCE was the highest at the 30 wt.% filler fraction.

Void fraction was calculated from the experimental density and theoretical density according to following equations:

*ρ_theoretical_* = 1/[(*W_filler_*/*ρ_filler_*) + (*W_matrix_*/*ρ_matrix_*)], (3)

and void fraction = (*ρ_theoretical_* − *ρ_experimental_*)/*ρ_theoretical_* (4)

where *W_filler_ and* *W_matrix_* are the weight fractions of the filler and the epoxy matrix, respectively, and *ρ_filler_*, and *ρ_matrix_* are the densities of the filler and the epoxy matrix, respectively. The void fraction was decreased because of surface modification and cross-linking.

## 4. Conclusions

In this study, a thermal conductive PCM was fabricated using PEG and BN. However, the interfacial adhesion between the BN and the PEG was poor, which hindered efficient heat conduction. PVA grafting and cross-linking the surface-modified BN with PEG improved the interfacial adhesion. As a result, the thermal conductivity of P-c-BN/PEG was 0.89 W/mK, which improved 286% compared to the raw PEG. In addition, the latent heat, which is an important property for PCMs, decreased after PVA grafting and cross-linking due to the decrease in the free volume of the polymer chain. However, this value is still suitable for PCM applications.

## Data Availability

The data presented in this study are available on request from the corresponding author.
